# Supplementary material for: Population size influences the type of nucleotide variations in humans
Source: BMC Genet. 2019 Dec 5;20:93. doi: 10.1186/s12863-019-0798-9 (PMC6894472; doi:10.1186/s12863-019-0798-9)
Supplement: Supplementary file 1 — Additional file 1: Figure S1. Correlation between effective population size (Ne) and the ratio AT→GC to GC→AT (b) estimated for homozygous SNVs from whole genomes of the 1000 Genomes Project. The relationship was highly significant (P < 10-6). Figure S2. Relationship between the effective population size (Ne) and the ratio of nucleotide changes within the same types (b): (A) within strong types i.e. C®G/G®C (B) within weak types i.e. A®T/T®A. The ratios were estimated using the high frequency SNVs (DAF > 0.9) belonging to 27 populations obtained from the 1000 genome Project. Figure S3. Relationship between the effective population size (Ne) and the ratio of nucleotide changes within the same types (b): (A) within strong types i.e. C→G/G→C (B) within weak types i.e. A→T/T→A. The ratios were estimated using the homozygous SNVs belonging to 126 populations obtained from the Simon Genome Project. Figure S4. Relationship between the effective population size (Ne) and the normalized ratio of AT®GC to GC®AT (b’) changes using equation 2 (see methods). We used A↔T and G↔C to normalize AT→GC and GC→AT changes respectively. (A) High frequency SNVs (DAF>0.9) and (B) Homozygous SNVs of the 1000 genome project (C) Homozygous SNVs from the Simons Genome Diversity project. The relationships were highly significant (P < 10-6). Figure S5. The relationship between effective population size (Ne) and the normalized ratio AT→GC/GC→AT (b) estimated for homozygous SNVs present in individual genomes belonging to 126 distinct populations of the world. This is very similar to Fig 4A except that the nucleotide diversities of non-Africans were 5% reduced while calculating Ne in order to neutralize the difference in mutation accumulation rates between Africans and non-Africans as reported recently. The correlation was highly significant (P < 10-6). [file 12863_2019_798_MOESM1_ESM.pdf]

Population size shapes the type of nucleotide variations in humans

Sankar Subramanian

Supplementary material

Supplementary Fig legends

Figure S1. Correlation between effective population size (N_e) and the ratio AT→GC to GC→AT (β) estimated for homozygous SNVs from whole genomes of the 1000 Genomes Project. The relationship was highly significant ($P < 10^{-6}$).

Figure S2. Relationship between the effective population size (N_e) and the ratio of nucleotide changes within the same types (β): **(A)** within strong types i.e. C→G/G→C **(B)** within weak types i.e. A→T/T→A. The ratios were estimated using the high frequency SNVs (DAF > 0.9) belonging to 27 populations obtained from the 1000 genome Project.

Figure S3. Relationship between the effective population size (N_e) and the ratio of nucleotide changes within the same types (β): **(A)** within strong types i.e. C→G/G→C **(B)** within weak types i.e. A→T/T→A. The ratios were estimated using the homozygous SNVs belonging to 126 populations obtained from the Simon Genome Project.

Figure S4. Relationship between the effective population size (N_e) and the normalized ratio of AT→GC to GC→AT (β') changes using equation 2 (see methods). We used A↔T and G↔C to normalize AT→GC and GC→AT changes respectively. **(A)** High frequency SNVs (DAF>0.9) and **(B)** Homozygous SNVs of the 1000 genome project **(C)** Homozygous SNVs from the Simons Genome Diversity project. The relationships were highly significant ($P < 10^{-6}$).

Figure S5. The relationship between effective population size (N_e) and the normalized ratio AT→GC/GC→AT (β) estimated for homozygous SNVs present in individual genomes

belonging to 126 distinct populations of the world. This is very similar to Fig 4A except that the nucleotide diversities of non-Africans were 5% reduced while calculating N_e in order to neutralize the difference in mutation accumulation rates between Africans and non-Africans as reported recently. The correlation was highly significant ($P < 10^{-6}$).

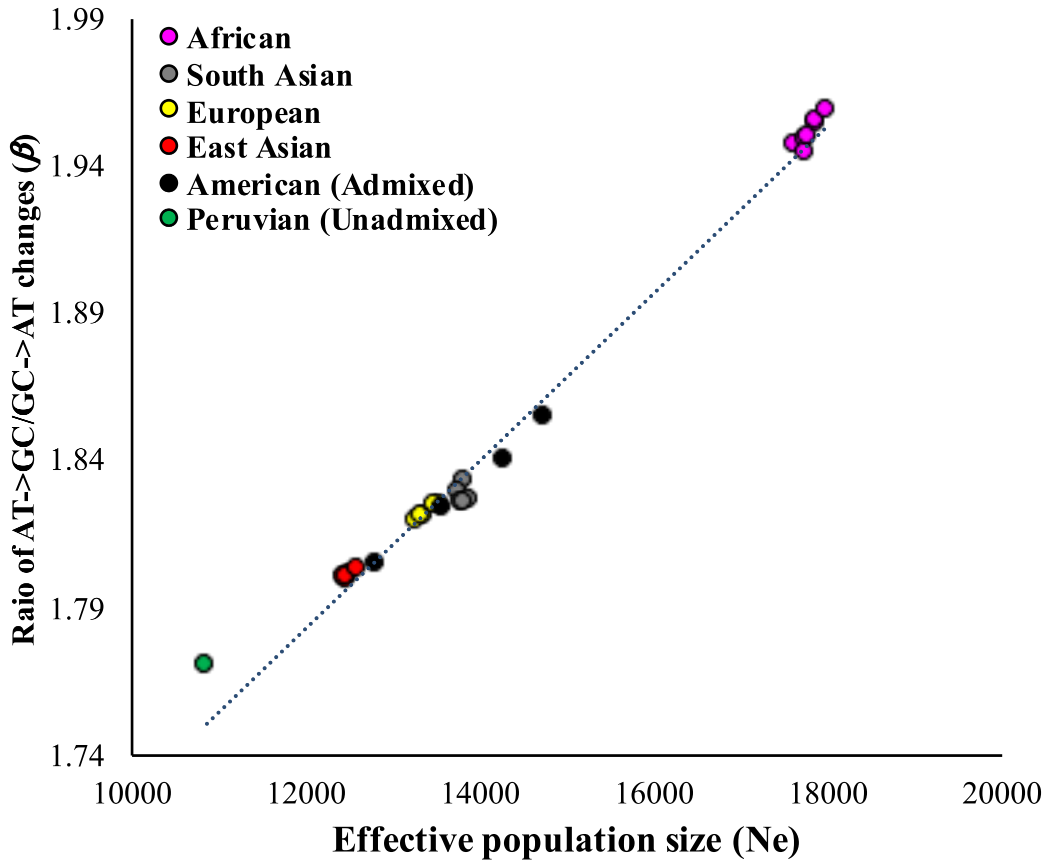

African
South Asian
European
East Asian
American (Admixed)
Peruvian (Unadmixed)
Raio of AT->GC/GC->AT changes (β)
1.99
1.94
1.89
1.84
1.79
1.74
10000
12000
14000
16000
18000
20000
Effective population size (Ne)

Figure S1

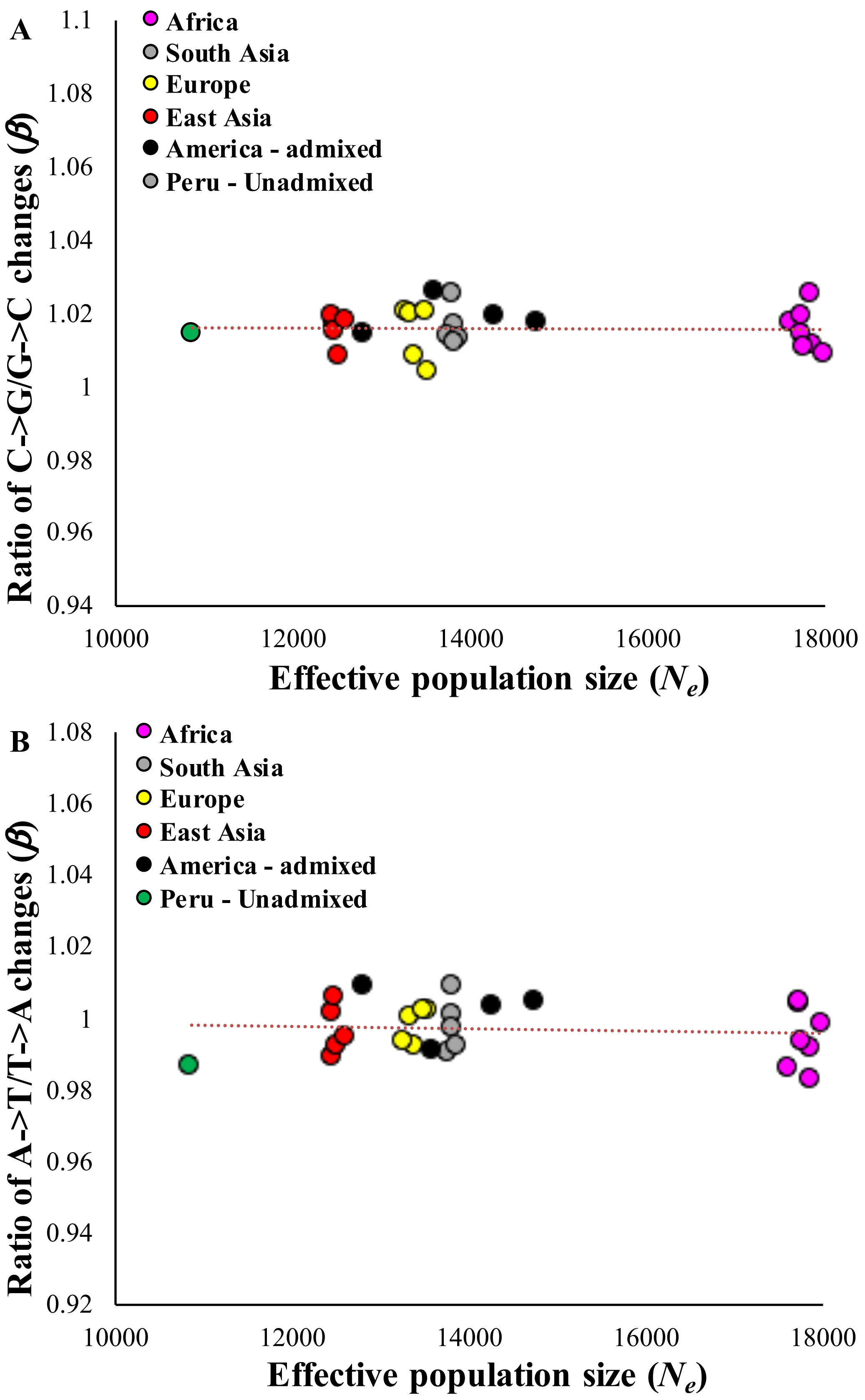
A
Africa
South Asia
Europe
East Asia
America - admixed
Peru - Unadmixed
Ratio of C->G/G->C changes (β)
1.1
1.08
1.06
1.04
1.02
1
0.98
0.96
0.94
10000
12000
14000
16000
18000
Effective population size (Ne)
B
Africa
South Asia
Europe
East Asia
America - admixed
Peru - Unadmixed
Ratio of A->T/T->A changes (β)
1.08
1.06
1.04
1.02
1
0.98
0.96
0.94
0.92
10000
12000
14000
16000
18000
Effective population size (Ne)

Figure S2

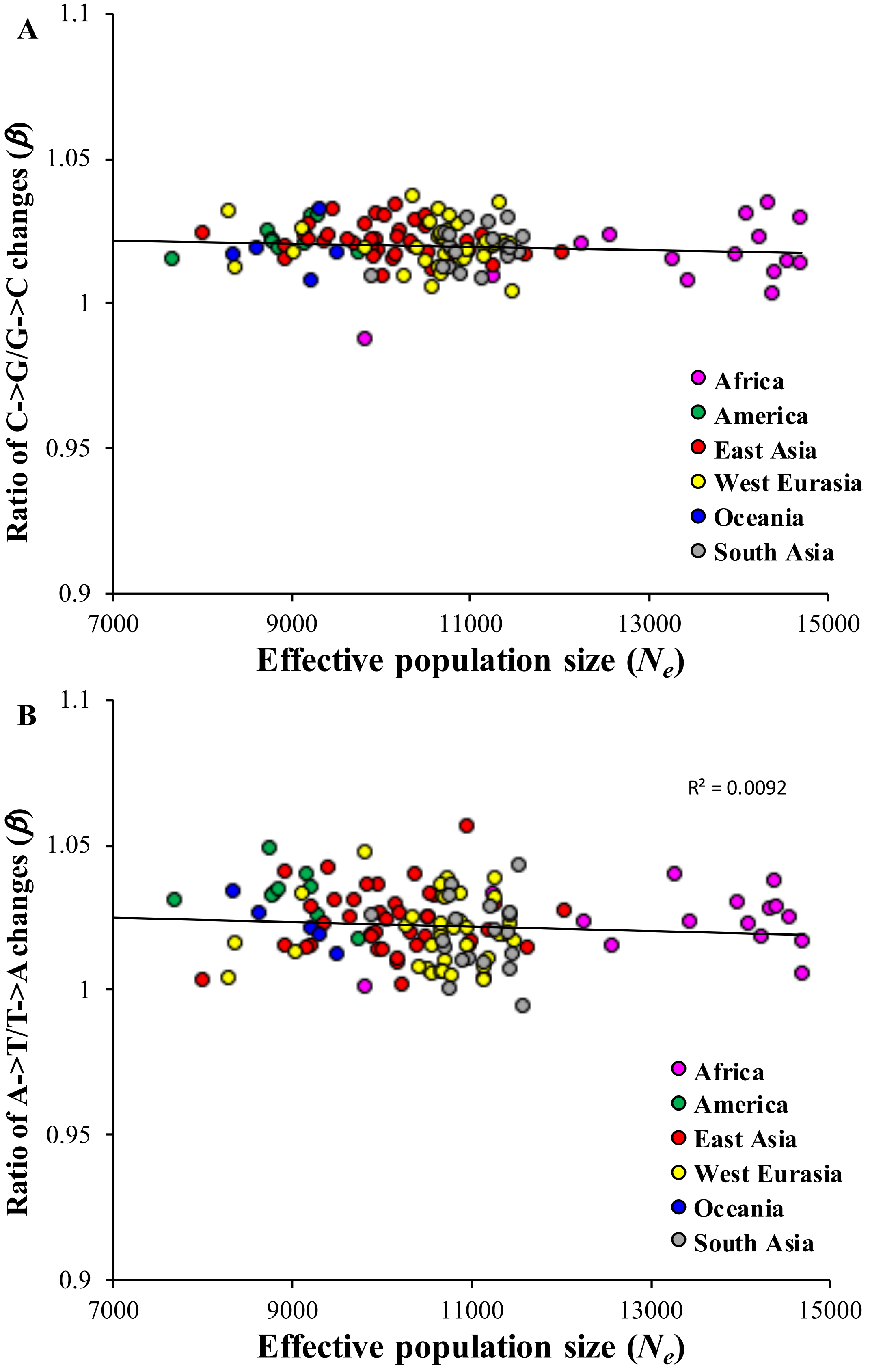
A
Ratio of C->G/G->C changes (β)
1.1
1.05
1
0.95
0.9
7000
9000
11000
13000
15000
Effective population size (N_e)
Africa
America
East Asia
West Eurasia
Oceania
South Asia
B
Ratio of A->T/T->A changes (β)
R² = 0.0092
1.1
1.05
1
0.95
0.9
7000
9000
11000
13000
15000
Effective population size (N_e)
Africa
America
East Asia
West Eurasia
Oceania
South Asia

Figure S3

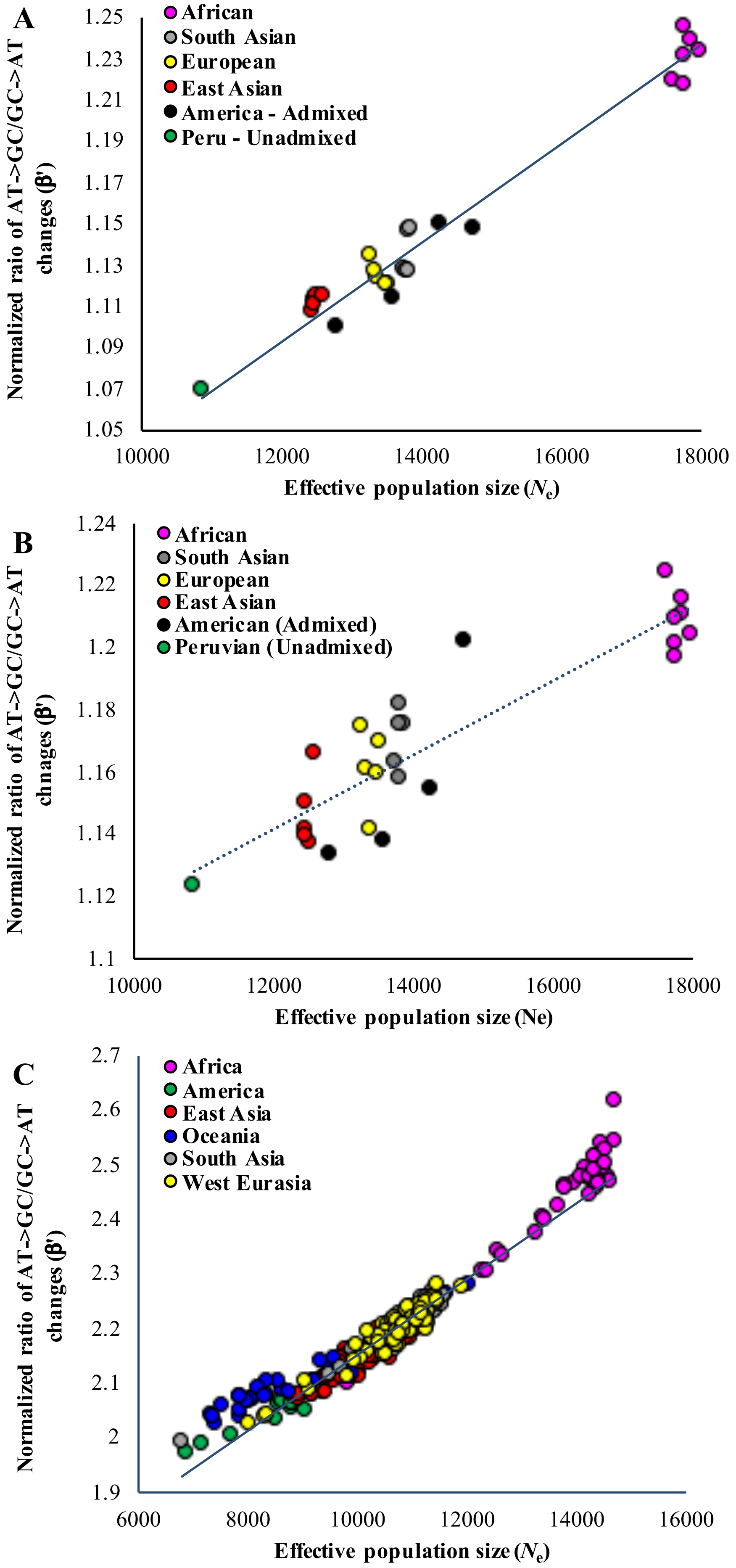
A
B
C
African
South Asian
European
East Asian
America - Admixed
Peru - Unadmixed
American (Admixed)
Peruvian (Unadmixed)
Africa
America
East Asia
Oceania
South Asia
West Eurasia
Normalized raio of AT->GC/GC->AT changes (β')
Normalized ratio of AT->GC/GC->AT chnages (β')
Normalized ratio of AT->GC/GC->AT changes (β')
Effective population size (N_e)
Effective population size (Ne)
1.25
1.23
1.21
1.19
1.17
1.15
1.13
1.11
1.09
1.07
1.05
1.24
1.22
1.2
1.18
1.16
1.14
1.12
1.1
2.7
2.6
2.5
2.4
2.3
2.2
2.1
2
1.9
6000
8000
10000
12000
14000
16000
18000

Figure S4

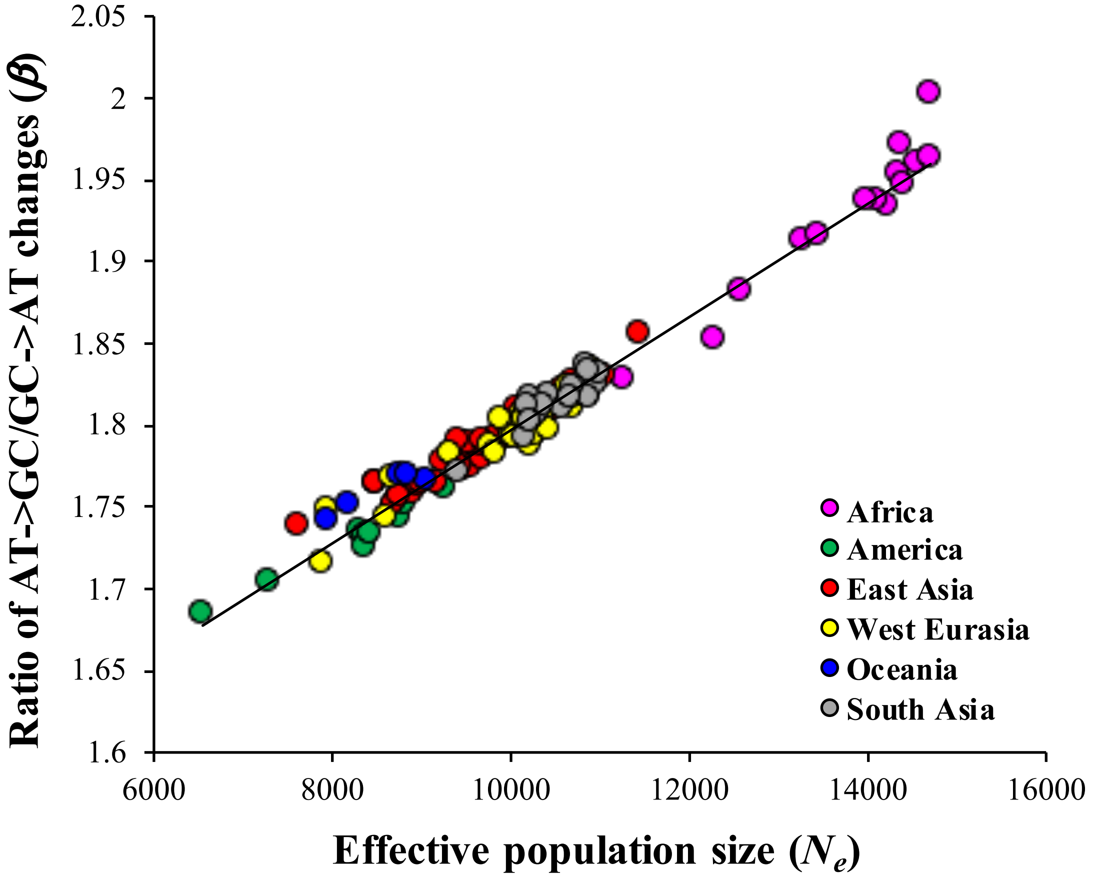
Ratio of AT->GC/GC->AT changes (β)
2.05
2
1.95
1.9
1.85
1.8
1.75
1.7
1.65
1.6
6000
8000
10000
12000
14000
16000
Effective population size (N_e)
Africa
America
East Asia
West Eurasia
Oceania
South Asia

Figure S5
